# Supplementary material for: Effects of High Cervical Spinal Cord Stimulation on Gait Disturbance and Dysarthropneumophonia in Parkinson’s Disease and Parkinson Variant of Multiple System Atrophy: A Case Series
Source: Brain Sci. 2022 Sep 10;12(9):1222. doi: 10.3390/brainsci12091222 (PMC9496748; doi:10.3390/brainsci12091222)
Supplement: Supplementary file 1 [file brainsci-12-01222-s001.zip › brainsci-1906854-supplementary.pdf]

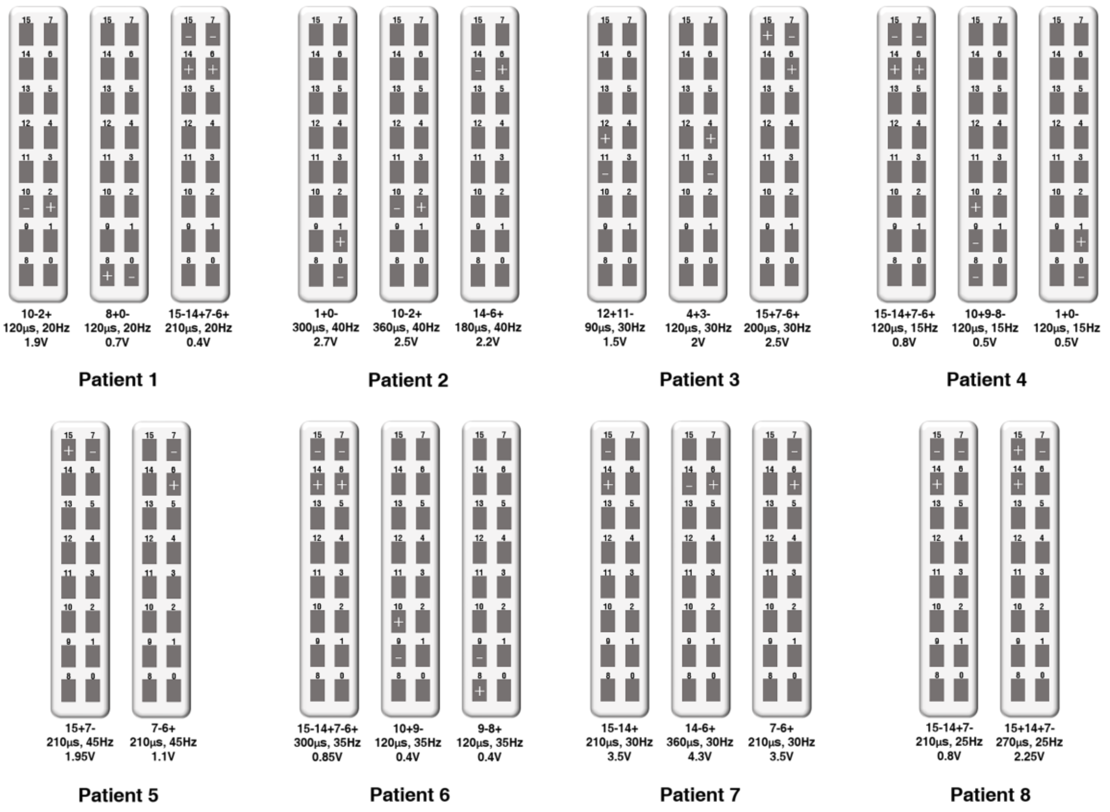

Supplementary Figure S1. Detailed simulation parameters in the upright body position determined for each patient at the latest follow-up period.
